# Association of chronic periodontitis with white blood cell and platelet count – A Case Control Study

**DOI:** 10.4317/jced.51292

**Published:** 2014-07-01

**Authors:** Balmuri P. Kumar, Tanya Khaitan, Pachigolla Ramaswamy, Pattipati Sreenivasulu, Ginjupally Uday, Ragha G. Velugubantla

**Affiliations:** 1 MDS, Reader. Department of Oral Medicine and Radiology, St. Joseph Dental College and Hospital. Duggirala, Eluru, Andhra Pradesh, India; 2 MDS, Post Graduate Student. Department of Oral Medicine and Radiology, St. Joseph Dental College and Hospital, Duggirala, Eluru, Andhra Pradesh, India; 3 MDS, Professor and Head. Department of Oral Medicine and Radiology. St. Joseph Dental College and Hospital, Duggirala, Eluru, Andhra Pradesh, India; 4 MDS, Senior lecturer. Department of Oral Medicine and Radiology, St. Joseph Dental College and Hospital, Duggirala, Eluru, Andhra Pradesh, India

## Abstract

Objectives:The objective of the study was to determine whether plasma levels of white blood corpuscles (WBCs) and platelets were altered in patients with chronic periodontitis compared to healthy controls. Study Design:A total of 120 subjects, 60 with chronic periodontitis and 60 with healthy periodontium of age group 30-50 years were selected for the study. Oral hygiene status and pocket probing depth were measured. During clinical evaluation, venous blood samples were taken to analyze the WBC and platelet counts. Statistical analysis was utilized to compare differences across various groups. Results:The WBC count was higher in patients with chronic periodontitis when compared with controls whereas the platelet count was lower in the case group. Conclusions:Elevated WBC count plays a key role in chronic periodontitis and in turn a risk factor for cardiovascular diseases. However, there is no significant role of platelets in periodontal infection even though it has a major role in atherogenesis.

** Key words:**Periodontitis, white blood cells, platelets, cardiovascular diseases.

## Introduction

Periodontal disease poses a significant challenge to the patients and the oral healthcare professionals. Studies conducted in India have shown that every second person above 35 years of age has periodontal pockets and 30% of total teeth extracted after 35 years of age are due to periodontal disease.

Non communicable diseases are taking an epidemic form and will be a major cause of death in developing countries by the year 2020. Over 29.8 million people have cardiovascular diseases in India. As a result of high prevalence of both cardiovascular disease and periodontal disease it has led to a hypothesis that these might be connected (1).

Increase in the number of white blood corpuscles [WBCs] is attributed to the increase of polymorphonuclear cells predominantly, which are key participants in the periodontal lesion and is also considered a strong inde-pendent predictor of future coronary heart disease (2,3). It has been hypothesized that platelets and leukocytes may be more sensitive to stimulation by periodontal pathogens and that activated platelets and leukocytes might contribute to increased atherothrombotic activity (4).

In light of above factors our study was aimed at determining, whether plasma levels of WBCs and platelets were altered in patients with chronic periodontitis compared to healthy controls.

## Material and Methods

The study was initiated after the protocol had been approved by the Institutional Committee of Research Ethics. All the subjects were being explained about the study and written informed consent were obtained.

A total of 120 subjects [60 cases and 60 controls] reporting to the department of Oral medicine and radiology, St. Joseph Dental College and Hospital, Eluru were recruited in the study. The patients enrolled in the study were belonging to the age group of 30-50 years. They were further divided into 4 subgroups:Group I [30 - 35 years of age], Group II [36 - 40 years of age], Group III [41 - 45 years of age] and Group IV [46 - 50 years of age] with 30 subjects [15 cases and 15 controls] in each group.

60 Subjects having ? 4 mm of pocket depth in more than 30% sites assessed in the oral cavity were considered as having chronic periodontitis (5). 60 subjects with a clinically healthy periodontium were included in the control group.

Patients with any systemic illness including diabetes, hypertension or any recent infection, pregnant women, those who were under nonsteroidal anti-inflammatory drugs [NSAIDs], antimicrobial drugs, mouthwashes or vitamin supplements for the past 3 months and all subjects with tobacco or alcohol habits were excluded from the study.

The armamentarium for the present study consisted of william’s periodontal probe, 5 ml syringe, vials containing ethylene diamine tetraacetic acid [EDTA], tourniquet, sterile cotton, surgical gloves.

The periodontal status of every individual was examined with a William’s periodontal probe according to Rus-sell’s periodontal index.

3 ml of venous blood was collected from all subjects by using routine venipuncture method and were stored in vials containing EDTA. The WBC and platelet counts were determined by using an automated cell counter wit-hin 24 hours after collection, at the hematology laboratory of the institution.

The data obtained was written in a tabulated manner and mean values and 95% confidence interval [CI] were calculated. Comparison of the 95% CI values of the WBC count and platelet count was performed using Mann-Whitney U test and Z test. The probability value [p value] less than 0.05 [p<0.05] was considered as statistically significant.

## Results

- Comparison of WBC and platelet count in case and control group: ( Table 1)

**Table 1:**
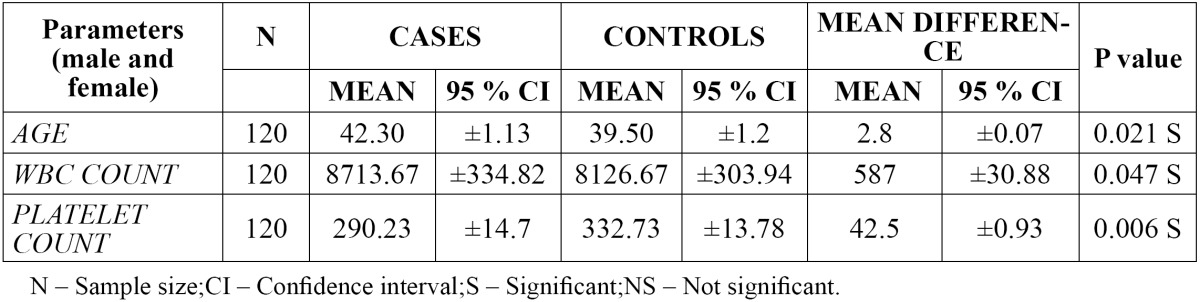
Comparison of WBC and platelet count in case and control group.

The mean WBC count was found to be higher in cases [8713.67] when compared to controls [8126.67] with a mean difference of 587. The 95% CI of WBC count was found to be ±334.82 in cases and ±303.94 in controls with a difference of ±30.88. The p value was found to be 0.047, which was statistically significant.

The mean platelet count was found to be lower in the case group [290.23] when compared to the control group [332.73] with a mean difference of 42.5. There was no much variation in the 95% CI of platelet count in the case group [±14.7] and the control group [±13.78] with a difference of ±0.93. The p value was found to be 0.006, which was statistically significant.

Comparison of WBC and platelet count in different age groups:

The mean value of WBC count was found to be higher [9453.33] in group II [36-40 years] of cases when compared to other groups with a mean difference of 1610.47. There was significant difference in the 95% CI values of WBC count in group II [36-40 yrs] and group IV [46–50 yrs] with a p value of 0.006 and 0.043 respectively ( Table 2).

**Table 2:**
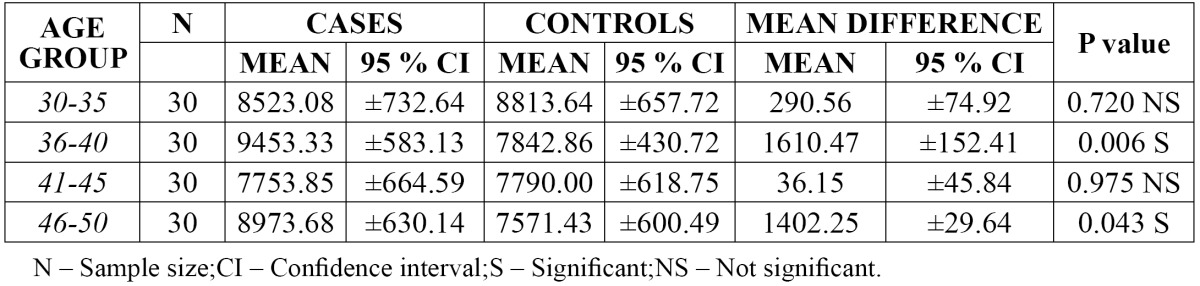
Comparison of WBC count in different age groups.

The mean value of platelet count was found to be higher [345.00] in group III [41-45 years] of controls when compared with other groups with a mean difference of 38.46. There was significant difference in the 95% CI values of platelet count in group I [30-35 years] when compared with other groups with a p value of 0.047 ( Table 3).

**Table 3:**
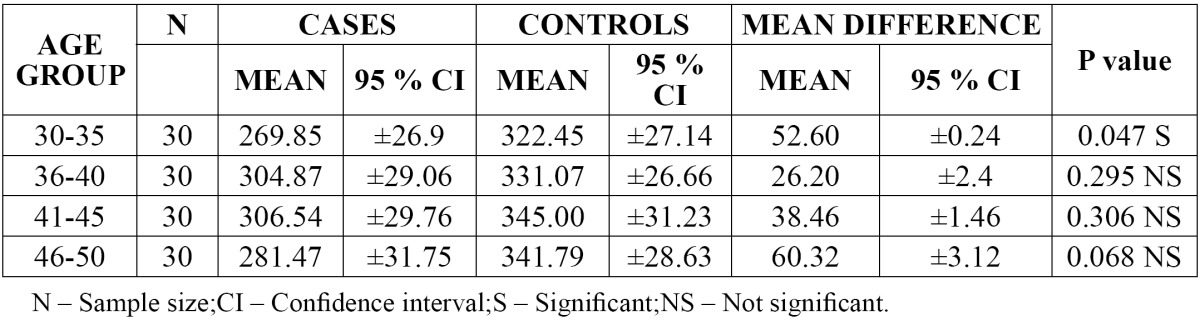
Comparison of platelet count in different age groups.

## Discussion

Leukocytes have a wide range of biological effects, some of which protect against vascular diseases while some are damaging (4). WBC count is associated with several cardiovascular disease risk factors. These findings have positive associations with body weight, systolic blood pressure, cigarette smoking, insulin levels and negative associations with high density lipoprotein cholesterol level, family income, alcohol consumption, physical acti-vity or fitness (6).

Periodontal infection leads to biofilm formation, inflammation and attachment loss. Continued inflammation results in signaling of fibroblasts and production of proinflammatory cytokines in the tissues. Antibodies specific to oral bacteria circulate in the peripheral blood. The acute-phase response becomes activated and C-reactive protein [CRP], fibrinogen and complement are produced both by local cells and within the liver. These proteins may further exacerbate the local inflammatory response and may affect the initiation or progression of systemic diseases like atherosclerosis. CRP is positively correlated to interleukin-6 and activates complement, which accounts for the uptake of low density lipoprotein [LDL] by macrophages. Activated WBCs reflect the inflammatory activity of atherosclerosis that perpetuates vascular injury and ischemia (7).

Ernst et al. have outlined three mechanisms by which leukocytes may contribute to microvascular injury and promote atherosclerosis. These include [a] pressure-dependent plugging of microvessels, [b] rheologic properties such as altered deformability and the formation of aggregates when provoked by a variety of stimuli, and [c] release of activated substances including oxygen-free radicals, proteolytic enzymes, and arachidonic acid metabolites (3).

Results of the present study indicate that individuals with periodontal infection have a higher WBC count com-pared to controls. This was in agreement with the studies conducted by Sambashivaiah, Yoshida et al, Rasheed and Rastogi et al (2-4,1). Renvert et al had also stated that recurrent acute coronary syndrome events are predicted by serum WBC counts and is a diagnosis of periodontitis (8).

Cigarette smoking is considered as the strongest predictor of attachment loss and bone loss (9). A number of studies indicate that the nicotine found in tobacco products triggers the overproduction of cytokines in the body due to lowered oxygen levels. Cytokines are signaling chemicals involved in the process of periodontal inflammation (10). But there are several changes in the blood like increased radical generation from peripheral neu-trophils, increased leukocyte count and reactive oxidants occurs which seem to be induced not only by cigarette but also by periodontitis (9). So in our study we had excluded subjects who were smokers.

Activated platelets regulate chemokine release by the monocytes in inflammatory lesions (4). Platelets are essential for primary hemostasis and endothelial repair, but also play a key role in atherogenesis and thrombus formation. Platelet count has been associated with vascular and non-vascular death and a recent meta-analysis showed that mean platelet volume is a predictor of cardiovascular risk (11).

The results of our study indicate that the group with chronic periodontitis has no much significant difference in the platelet count when compared to healthy group which suggests that platelets have no significant role in periodontal infection.

## Conclusions

Thus our study concludes that the chronic periodontitis has an association with increased WBC count and in turn is a risk factor for cardiovascular diseases. There is no significant role of platelets in periodontitis although it has major role in atherosclerosis.
